# Cachectic muscle wasting in acute myeloid leukaemia: a sleeping giant with dire clinical consequences

**DOI:** 10.1002/jcsm.12880

**Published:** 2021-12-08

**Authors:** Dean G. Campelj, Cara A. Timpani, Emma Rybalka

**Affiliations:** ^1^ Institute for Health and Sport (IHeS) Victoria University Melbourne Victoria Australia; ^2^ Australian Institute for Musculoskeletal Science (AIMSS) St Albans Victoria Australia; ^3^ Department of Medicine—Western Health, Melbourne Medical School The University of Melbourne Melbourne Victoria Australia

**Keywords:** Acute myeloid leukaemia, Skeletal muscle, Cachexia, Chemotherapy, Cancer, Myopathy, Risk stratification

## Abstract

Acute myeloid leukaemia (AML) is a haematological malignancy with poor survival odds, particularly in the older (>65 years) population, in whom it is most prevalent. Treatment consists of induction and consolidation chemotherapy to remit the cancer followed by potentially curative haematopoietic cell transplantation. These intense treatments are debilitating and increase the risk of mortality. Patient stratification is used to mitigate this risk and considers a variety of factors, including body mass, to determine whether a patient is suitable for any or all treatment options. Skeletal muscle mass, the primary constituent of the body lean mass, may be a better predictor of patient suitability for, and outcomes of, AML treatment. Yet skeletal muscle is compromised by a variety of factors associated with AML and its clinical treatment consistent with cachexia, a life‐threatening body wasting syndrome. Cachectic muscle wasting is associated with both cancer and anticancer chemotherapy. Although not traditionally associated with haematological cancers, cachexia is observed in AML and can have dire consequences. In this review, we discuss the importance of addressing skeletal muscle mass and cachexia within the AML clinical landscape in view of improving survivability of this disease.

## Introduction

The most common acute leukaemia in adults, acute myeloid leukaemia (AML), is a malignant disorder of haematopoietic stem cells resulting in impaired production of the myeloid blood cell lineage.^1^ AML is initially treated with intense chemotherapy induction regimens, followed by haematopoietic cell transplantation (HCT) to achieve complete remission (CR). While these strategies have had some success in younger cohorts, their clinical utility is constrained in older adults: the intensity and cytotoxicity of the chemotherapy used in the induction regimen and HCT conditioning programme enhances the risk of multi‐organ toxicity, promoting morbidity and mortality.^2^ This aspect reduces the curability of AML in older patients and directly contributes to the higher mortality in >65‐year‐old patients.^2^ Thus, it is important to pursue novel ideas to optimize current treatment and risk management strategies to improve outcomes for AML patients.

One emerging consideration in patient risk stratification for AML is skeletal muscle mass (MM). Skeletal muscle wasting and dysfunction (referred to herein as myopathy) is a consequence of many cancers and is an event synonymous with the induction of the body wasting syndrome, cachexia. Cancer‐associated cachexia is considered to compound with sarcopenia, the age‐related loss of MM and function,^3^ and malnutrition^4^ in older cancer patients resulting in dire mortality rates. Cachexia affects a staggering 50–80% of cancer patients, and up to 80% of those who manifest cachexia will die.^5^ Recently, it has emerged that cachexia is synergistically driven by both cancer‐related factors and anticancer chemotherapy treatments.^6^ Solid tumours, in particular, release many signalling factors that drive myopathy. Because cachexia is most prevalent in solid tumours associated with the gastrointestinal (GI) system (including accessory organs) and lungs, disruption of systemic nutrient delivery (i.e. amino acids and oxygen) may be a contributing factor to skeletal muscle wasting, albeit parenteral nutrition does little to attenuate cachexia.^6^ However, in a clinical setting where patients are quickly treated for diagnosed cancers while cachexia is emerging, it is difficult to separate out the contribution made by cancer factors and anticancer treatment. Challenging the current dogma, the burgeoning field of chemotherapy‐induced cachexia research indicates that chemotherapy both independently drives skeletal myopathy and exacerbates cancer‐associated myopathy.^7^ Thus, chemotherapy appears to have a more prominent role in cachexia than originally considered. For haematological cancers, such as AML where intense chemotherapy is the primary (or only) treatment modality, the scope for severe chemotherapy‐induced cachexia is profound. This poses serious consequences for AML patients: skeletal MM has emerged as a prominent prognostic factor for survivability, not only in older adults but also for patients diagnosed at any age.^8^, ^9^ Unfortunately, there is currently little emphasis on the impact of skeletal muscle during patient risk stratification in AML, despite its potential as a factor to optimize clinical decision making and for therapeutic intervention.

This review will provide a current account of the evidence surrounding the impact of AML, and its treatments, on skeletal muscle and highlight the potential for skeletal muscle health to be clinically utilized in patient risk stratification and clinical decision making in AML.

## Overview of the clinical landscape of acute myeloid leukaemia

Acute myeloid leukaemia is characterized by uncontrolled clonal expansion and differentiation arrest of haematopoietic stem cells, whereby the hyper‐production of immature myeloid progenitors replaces homeostatic haematopoiesis.^1^ Specifically, AML suppresses the production of essential blood cells from the myeloid lineage (e.g. erythrocytes, platelets, and granulocytes), leading to anaemia, haemorrhage, and infection, respectively.^1^ AML has a 5 year overall survival (OS) of ~29%.^10^ However, this statistic is negatively skewed by age with adults >65 years old having a 5 year OS of only ~8%.^10^ These statistics are even more grim considering that (i) the median age of AML diagnosis is 68 years old^11^; (ii) AML incidence is three‐fold higher in adults >65 years old^10^; and (iii) over the past decade, the prevalence of AML has increased proportionately with the ageing population.^12^ This highlights age as a key prognostic indicator. In 2021, there is expected to be 20 240 new AML cases and 11 400 associated deaths in the USA alone,^13^ which projects AML to have the greatest mortality to incidence ratio of all blood cancers.^13^ The average cost of AML treatment in the USA is ~$200 000 USD per chemotherapy induction regimen and ~$330 000 USD per HCT, which is attributed to extensive inpatient hospitalization.^14^ These figures exclude the costs associated with multiple chemotherapy cycles and HCTs, and relapse. Thus, the true disease and economic burden on patients and subsidizing healthcare systems is substantial and will only increase with the ageing population. Because AML has a median survival time of 8.5 months in the USA,^11^ the initial treatment period is crucial to successful treatment outcomes. This is particularly true because, despite several promising novel treatments for AML being trialled for younger patients, there has been a distinct lack of progression in treatments targeting the predominant and most at‐risk older adult population.

Since the 1970s, AML has been clinically treated with conventional chemotherapy, primarily the ‘3 + 7’ induction regimen, which involves 3 days of an anthracycline (daunorubicin, epirubicin, or idarubicin) concurrent with 7 days of the antimetabolite, cytarabine.^1^ The ‘3 + 7’ induction regimen can be repeated multiple times before achieving CR. Future treatment decision making is then dependent upon risk stratification, which considers a suite of prognostic factors for relapse post‐induction (e.g. cytogenetics and molecular mutations; for extensive review, see Papaemmanuil *et al*.^15^). AML patients with favourable risk profiles typically receive further chemotherapy as a ‘consolidation’ treatment, such as high‐dose cytarabine, to maintain CR and reduce the potential of minimal residual disease—a major cause of relapse in AML.^1^ However, most AML patients present with intermediate or adverse risk profiles,^15^ which requires allogeneic HCT, the only current curative strategy for AML.

Preparation for HCT is initiated through an intense conditioning programme, typically utilizing a chemoradiation strategy, such as cyclophosphamide, alongside high‐dose busulfan or total body irradiation.^16^ These induce myeloablation but are associated with severe toxicity and co‐morbidity. Nevertheless, it is an essential constituent of HCT, which confers a significant survival advantage for eligible patients.^17^ However, in older adults, the utilization of HCT is limited. Only 10% of AML patients >65 years old are prescribed with HCT due to the high risk of graft vs. host disease (GvHD) and transplant‐related mortality.^18^ Reduced‐intensity conditioning programmes coupled with improved supportive care and more accurate HLA‐type matching have increased the risk/reward ratio of HCT prescription in older AML patients somewhat.^18^ While an increased risk of severe GvHD remains in this patient cohort, the benefits (i.e. reduced risk of relapse and increased OS) are profound.^19^ Thus, it is imperative to investigate new strategies to increase AML patient eligibility for HCT to achieve a more sustainable CR. In particular, the ongoing optimization of patient risk stratification is essential, which requires novel ideas to better guide current therapeutic strategies and decision making for AML.

## The impact of body composition on successful acute myeloid leukaemia treatment: a novel target for risk stratification?

Research arising over the past decade may have disingenuously overlooked the screening potential of anthropometry parameters in AML risk stratification. This is likely due to the lack of consensus surrounding the prognostic value of crude body composition indicator, body mass index (BMI), at diagnosis.^20^, ^21^ The key issue is that BMI does not accurately measure adiposity nor MM,^22^ allowing for an ‘obesity paradox’ in cancer patients where the relationship between BMI and mortality does not adhere to its typical J‐curve.^23^ Instead, patients classified as overweight or obese (BMI > 25 kg/m^2^) often demonstrate better survivability.^24^ An exception to this paradigm, younger AML patients (<65 years old) categorized as overweight or obese display worse OS and GvHD‐free survival compared with patients with lower BMI scores (<25 kg/m^2^).^25^ However, these adverse prognostic findings are suggested to be partly dependent on dose‐capping at a body surface area (BSA) of 2 m^2^, which occurs in ~40% of obese cancer patients and is associated with reduced therapeutic efficacy of chemotherapeutic agents (CAs).^26^ Optimal dose selection is integral in AML treatment decision making and is based on risk stratification to ensure therapeutic efficacy while limiting drug‐related toxicity (DRT) and non‐disease mortality.^27^ To avoid the issues surrounding dose‐capping, additional studies evaluated chemotherapy dosing based on actual body mass (BM) rather than BSA with mixed results.^28^, ^29^, ^30^ This suggests that dose‐selection strategies require a more accurate body composition normalization measure to overcome the ‘obesity paradox’ and mitigate DRT. Historically, dose selection, derived from the classic BSA formula,^31^ was based on the theory that larger patients have a higher capacity for drug clearance and thus require higher doses to reach equivalent bioavailable drug concentrations compared with smaller patients.^32^ However, the clinical utility of BSA in the context of CA dosing circumvented the developing understanding of individual drug pharmacokinetics: BSA normalization is unable to account for the variability in plasma drug concentrations and clearance,^33^ highlighting a need for alternative dose‐selection strategies. Lean BM (LBM), that is, the sum of body water, total body protein, carbohydrates, non‐fat lipids, and soft tissue mineral,^22^ is a promising alternative to BSA for chemotherapy dose‐related decision making.^34^ BSA does not account for the large and unpredictable role of fat mass relative to BM and is poorly correlated with LBM in cancer patients,^35^ while LBM can account for a significant portion of the variation in the clearance of CAs, for example, the anthracycline epirubicin.^36^ Thus, increasing the accuracy of body composition assessment is a promising strategy to improve risk stratification processes and mitigate DRT while improving patient survivability in populations in whom changes to body composition are masked by total BM.

Body composition assessments are an emerging frontier of personalized medicine and risk stratification in the oncological setting.^4^ They are particularly important in the at‐risk older adult AML cohort, a population also at high risk for sarcopenia, which is poorly correlated with BMI classification.^37^ However, BMI does have some prognostic value in the geriatric AML patient population: lower BMI scores in older adults (>60 years old) are associated with reduced OS and GvHD‐free survival and are exacerbated in patients classified as underweight (BMI < 18.5 kg/m^2^) compared with normal weight (BMI 18.5–25 kg/m^2^).^38^ These data do not break the constraints surrounding current risk stratification practice, with underweight geriatric populations routinely flagged as high risk for DRT in geriatric assessments, for example, Karnofsky Performance Scale, evaluating suitability to receive intense chemotherapy.^39^ A complementary body composition assessment modality is the skeletal muscle index (SMI), the gold standard for detecting changes in skeletal MM ascertained using computed tomography (CT) scans to quantitate total abdominal muscle area corrected for height (cm^2^/m^2^) at the third lumbar (L3) level.^40^ Recently, reference ranges of the total abdominal muscle area were published, with cut‐off points determining low MM to range between 36.54–45.2 and 30.21–36.05 cm^2^/m^2^ in healthy individuals and 36–43.2 and 29–34.9 cm^2^/m^2^ in cancer patients, for men and women, respectively.^41^ For cancer patients, and consistent with the ‘obesity paradox’, a similar ‘BMI paradox’ exists: low MM, that is, myopenia (any age) or sarcopenia (specifically in older age), can be hidden in patients classified as normal or overweight (BMI 18.5–29.9 kg/m^2^), and low MM alongside high adiposity, that is, sarcopenic obesity, can be hidden in patients classified as obese (BMI > 30 kg/m^2^).^42^ Accordingly, Martin *et al*. stratified myopenia cut‐off values in lung and GIT cancer patients through accounting for BM‐related variance, with criteria proposed: <41 cm^2^/m^2^ for women; <43 cm^2^/m^2^ (BMI < 25 kg/m^2^) and <53 cm^2^/m^2^ (BMI ≥ 25 kg/m^2^) for men.^43^ While these cut‐off values for CT‐derived SMI and BMI in cancer‐specific subpopulations are the most refined to date, there is further investigation required to cultivate tertile cut‐off ranges that are more sensitive to the changes in BMI and CT‐derived SMI typically observed in large and diverse population‐based multicentre trials. Future research should also pursue the refinement of these myopenia/sarcopenia and sarcopenic obesity cut‐off ranges specific to the cancer type and especially for AML.

Despite being commonly undetected because of the clinical reliance on BMI, myopenia and sarcopenia at the time of cancer diagnosis are associated with increased risk of DRT from, and consequently poor adherence to, anticancer treatment.^42^, ^44^ This instigates a vicious wasting cycle because chemotherapy exacerbates myopenia, thus potentiating the risk of mortality.^45^ While sarcopenia is central to these findings, it is known that this condition is typically present alongside other co‐morbidities, with recent data suggesting there is a nine‐fold increase in the likelihood to have a Charlson Comorbidity Index >2 in sarcopenic individuals.^46^ These co‐morbidities include low nutrition status, diabetes, cardiovascular disease, kidney disease, and liver disease, which may all contribute to poorer treatment outcomes and survivability in AML.^47^ There are less empirical data available regarding the connection between myopenia and co‐morbidities, predominately due to the lack of consensus of a definition of skeletal muscle wasting that accounts for conditions across all ages, not just the older population.^48^ Specific to AML, myopenia and sarcopenia pre‐diagnosis are prognostic of poorer survivability.^8^, ^9^ However, the outlook for the older AML cohort is particularly devastating: the 3 year OS for sarcopenic patients is 0% compared with 49% for non‐sarcopenic patients.^8^ Interestingly, sarcopenia, characterized solely by low SMI, was associated with reduced muscle function in AML patients^49^despite the poor correlation of SMI with functional measurements,^50^ leading to more questions rather than answers. These findings highlight the importance of skeletal MM at diagnosis and provoke questions regarding the potential interplay between myopenia/sarcopenia and fellow wasting condition, cachexia, at other important stages of AML treatment such as before and after HCT, which could have serious implications for AML patients of any age.

**Figure 1:**
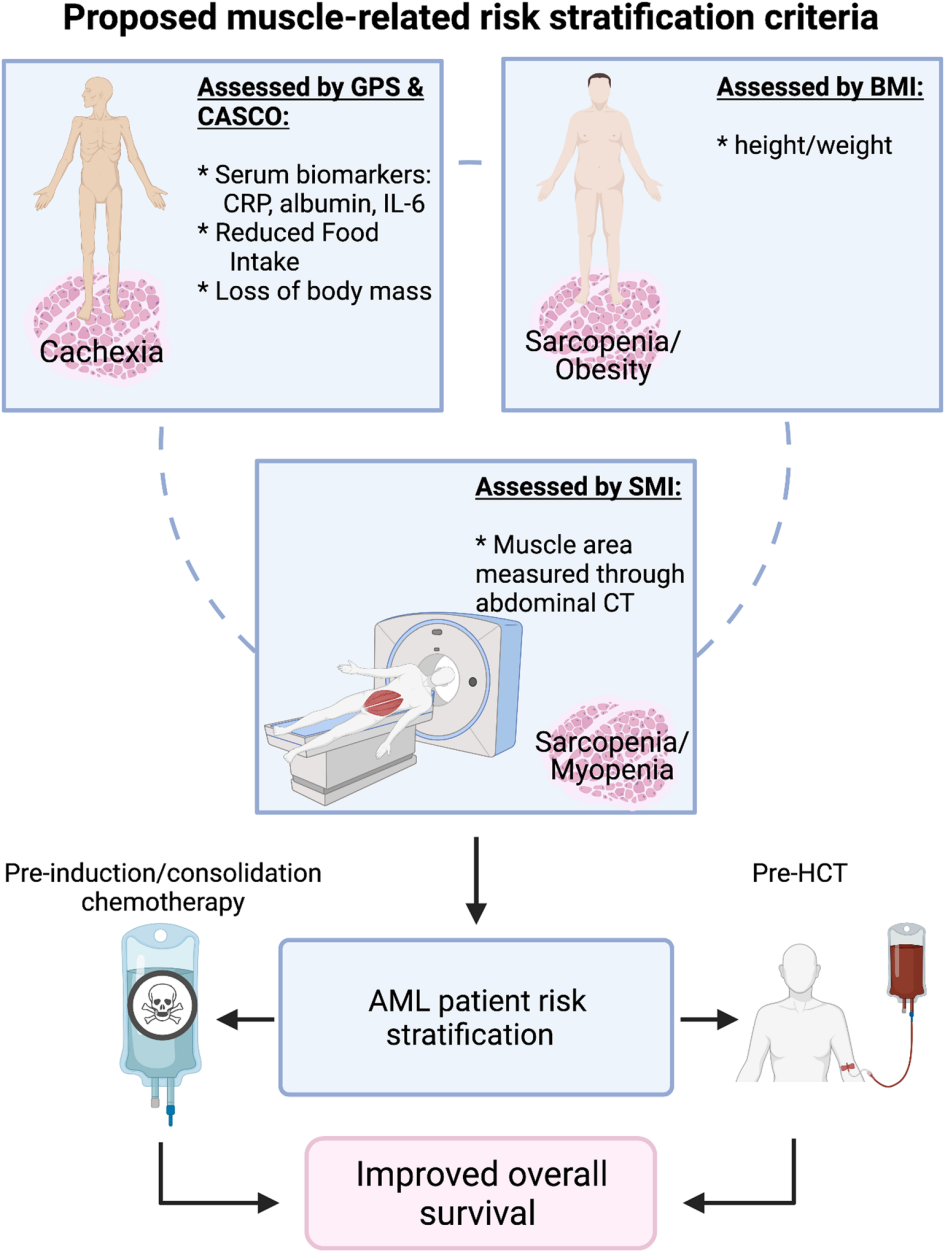
Proposed muscle‐related risk stratification criteria in acute myeloid leukaemia (AML). Improved risk stratification criteria for AML could consider, measure, and utilize skeletal muscle mass because muscle loss is correlated with higher morbidity and mortality rates. Patients could be stratified through the identification of (i) cachexia by the CAchexia SCOre (CASCO), which utilizes pro‐inflammatory status [measured by interleukin‐6 (IL‐6)], and the Glasgow prognostic score (GPS), which measures serum C‐reactive protein (CRP) and albumin; (ii) sarcopenia/obesity by the body mass index (BMI) using the rudimentary measures of patient height and weight; or (iii) myopenia/sarcopenia by the skeletal muscle index (SMI), the gold standard assessment of skeletal muscle mass by abdominal computed tomography (CT). This could occur before, during, and after AML treatment to determine acceptable chemotherapy exposure (e.g. type of anthracycline, dose, and number of consolidation cycles) and eligibility for haematopoietic cell transplantation (HCT) and increase overall survival.

Cancer‐associated cachexia is defined as the loss of BM alongside the depletion of skeletal MM with, or without, loss of fat mass.^51^ Cachexia is clinically characterized by (i) weight loss of >5% over a 6 month period; (ii) BMI < 20 kg/m^2^ and body weight loss of 2%; or (iii) an appendicular SMI consistent with sarcopenia and weight loss >2% over the past 6 months.^51^ Importantly, skeletal MM loss is purported as the quintessential prognostic factor in cachexia, particularly when conceding the impact of the ‘obesity paradox’.^43^ Indeed, Roeland *et al*. highlighted that monitoring BM changes may not be sensitive enough to capture cachexia‐induced skeletal MM loss.^52^ The diagnosis of cachexia is a dynamic continuum of staging from pre‐cachexia to cachexia to refractory cachexia, and progression through each stage is associated with increased morbidity and mortality risk.^51^ Cachexia comprises multifactorial underlying systemic issues, including anorexia, chronic inflammation, and altered energy metabolism.^6^ Collectively, these factors perturb the nutritional status of cachectic patients. This is acknowledged within the definition of cachexia, that is, that BM loss cannot be fully reversed by conventional nutritional support.^51^ Further, cachectic muscle wasting is accompanied by progressive dysfunction, which reduces exercise tolerance, physical performance, ambulatory capacity, and ability to undertake activities of daily living, which reduce patient quality of life (QoL). Cachexia is not typically considered as part of risk stratification in AML nor other haematological malignancies, despite its prevalence in these cancers and the deleterious impact it has in other cancers.^6^ Although confirmatory data from multicentre studies with larger sample sizes are required, Keng *et al*. suggest that AML patients experience a median BM loss of ~6% from diagnosis to CR attainment, which is consistent with the definition of cachexia.^53^ Moreover, the loss of BM from AML diagnosis to the commencement of HCT is associated with reduced OS and GvHD‐free survival.^54^ These findings build on emerging evidence that lower BMI scores before HCT are associated with increased risk of relapse and mortality,^55^ where the degree of wasting corresponds with the risk of negative treatment outcomes.^56^ Further, and perhaps more importantly, pre‐HCT abdominal CT scans measuring myopenia/sarcopenia‐defined MM loss have been identified as a powerful prognostic factor in determining non‐relapse mortality after HCT.^57^ However, neither skeletal muscle nor BM loss during treatment is considered as a risk factor for HCT eligibility. Remarkably, only a pre‐HCT BMI score >35 is included in the HCT co‐morbidity index,^58^ which typically identifies a morbidly obese patient.^23^ It is also important to consider pre‐HCT BM loss for risk stratification purposes. Weight loss history before HCT is a significant predictor of patient deconditioning,^59^ and superfluous weight loss after HCT increases non‐relapse mortality risk.^60^ Thus, developing screening strategies for indicators of patient susceptibility for BM (particularly lean mass) loss during treatment would enrich risk stratification guidelines at multiple treatment stages (i.e. during induction, consolidation, and/or HCT; *Figure* 1). Further, understanding the factors that contribute to cachexia induction in the AML setting is essential for identifying strategies to mitigate the influence of cachexia on poor treatment outcomes.

## An opportunity to waste: exploring the multifactorial contributors to skeletal myopathy in acute myeloid leukaemia

**Figure 2:**
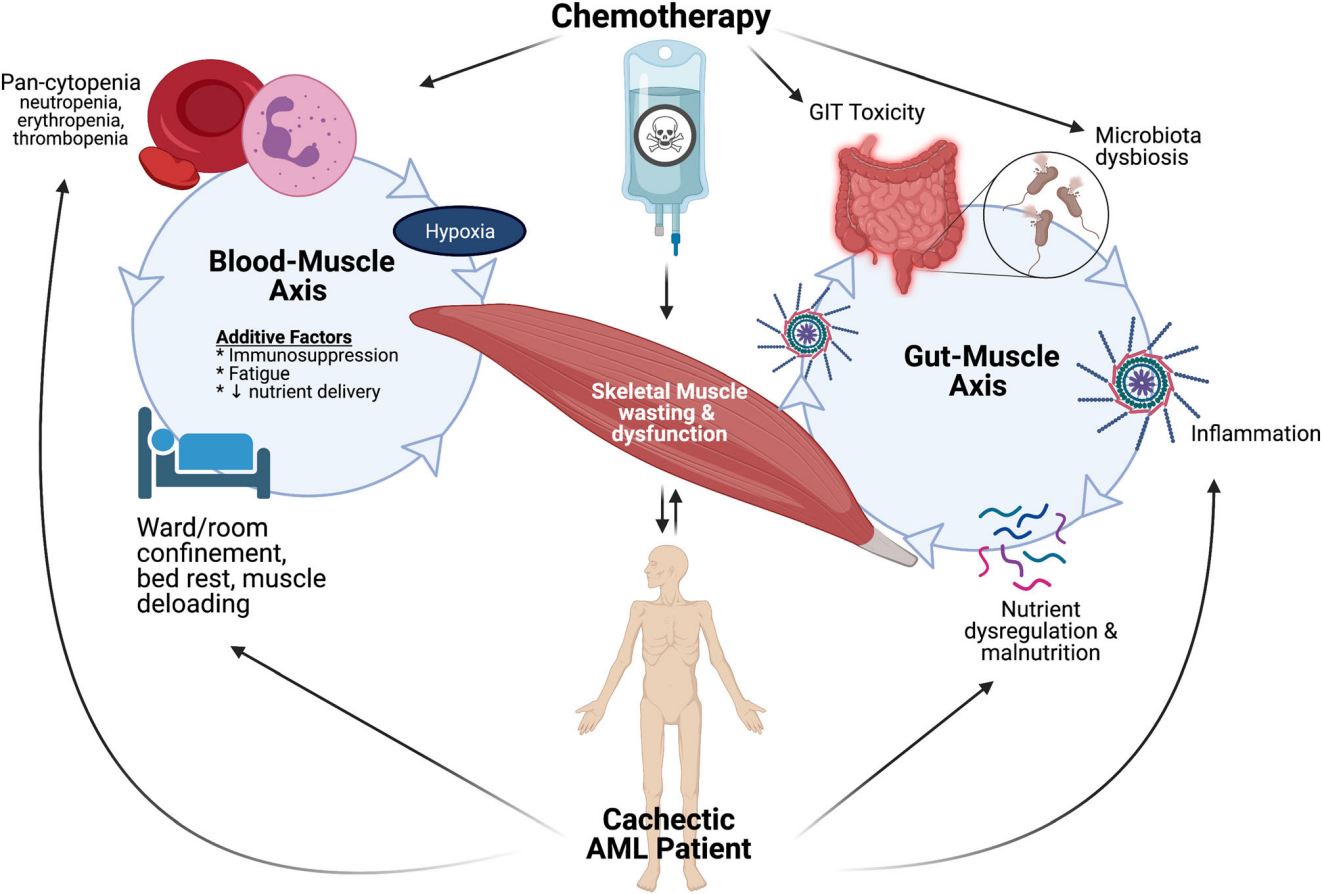
Potential drivers of muscle mass loss in acute myeloid leukaemia (AML). Chemotherapy administration has been shown to directly induce skeletal muscle wasting and dysfunction. This can be exacerbated by the effect of chemotherapy on the gastrointestinal tract (GIT), which dysregulates motility and microbiota composition and compromises the intestinal barrier. Collectively, these symptoms reduce nutrient absorption and nutrient status signalling through the microbiome and increase the risk of infection and passage of inflammatory mediators. Furthermore, chemotherapy extirpates blood cells causing hypoxia and supressing the immune response. Consequently, patients are incapacitated, because of both fatigue and risk of infection, respectively. Ultimately, processes that maintain muscle mass and function are compromised, and muscle damage and wasting is potentiated. The outcome is cachexia, which further drives muscle wasting and dysfunction through dysregulated signalling, malnutrition, and muscle de‐loading.

Encompassing ~40% of total BM, skeletal muscle is essential for locomotion, mastication, swallowing, and breathing, highlighting its necessity for undertaking activities of daily living and preserving QoL.^61^ Skeletal MM is controlled through balancing protein synthesis and degradation (i.e. muscle turnover) and can be influenced by many hypertrophic and atrophic factors.^62^ Skeletal muscle also regulates basal metabolic rate. It is both a ‘sink’ for blood glucose disposal (stored as glycogen) and a reservoir for amino acids: both can be metabolized for energy production and are important signals for muscle growth.^61^, ^63^ While these stores are maintained by regulatory homeostatic processes, under severe metabolic stress caused by wasting conditions like cachexia, muscle protein can become depleted through catabolism. Consequently, metabolism is dysregulated, contributing to debilitating dysfunction. Discovering the underlying mechanisms that drive skeletal myopathy in cancer cachexia has received attention, predominantly through studies in rodent models or human patients with solid tumours.^64^ A host of potential drivers of the cachectic condition have been illuminated (summarized in *Figure* 2). However, there is limited understanding concerning the contributing factors that drive muscle cachexia in haematological malignancies, such as AML.

### Chemotherapy‐induced myopathy

Over the past decade, the impact of CAs on skeletal muscle has become increasingly evident as recently reviewed by us.^7^ The anthracycline class of CAs have been at the forefront of this research, primarily using doxorubicin, an analogue of daunorubicin/epirubicin/idarubicin used interchangeably in the ‘3 + 7’ AML induction regimen. Anthracyclines are widely known to induce cardiomyopathy,^65^ a key risk factor associated with their limited clinical utility in AML treatment.^66^ It is also well documented that doxorubicin induces skeletal myopathy as observed in cell culture, rodent models, and humans, with the mechanisms extensively investigated.^7^ Briefly, it has been shown that doxorubicin (i) promotes mitochondrial toxicity through hijacking complex I redox cycling and (ii) amplifies reactive oxygen species production. Together, these can impair mitochondrial respiration and cause oxidative stress/damage.^67^ Oxidative stress promotes skeletal muscle catabolism by up‐regulating proteolytic pathways that degrade myofibrillar proteins including myosin and actin, resulting in contractile apparatus dysfunction.^67^ Daunorubicin also reduces myosin and actin expression and disrupts sarcomeric organization in C2C12 myotubes.^68^ While these data highlight the deleterious impact of certain anthracyclines on the quality and quantity of skeletal muscle, further studies are required to contextualize their impact in AML treatment because daunorubicin, epirubicin, and idarubicin differ in their chemical structure and may exert different degrees of toxicity on the muscular system.

It is important to acknowledge that not all CAs exert comparable effects on skeletal muscle nor have they all been evaluated, indicating a significant knowledge gap. In AML, there is limited understanding of the effect of cytarabine, the other constituent of the ‘3 + 7’ chemotherapy induction regimen, on skeletal muscle. Amrute‐Nayak *et al*. is the only study of note to investigate this, and no impact of cytarabine was reported.^68^ However, the authors did not justify dose selection, and given that CAs elicit skeletal myopathy in a dose‐dependent manner,^7^ dose‐escalation studies would be beneficial. Cytarabine is an antimetabolite like 5‐fluorouracil, an established potent activator of cell stress signalling factor, mitogen‐associated protein kinase (MAPK).^69^ During oxidative stress, MAPK activation, particularly p38 MAPK, promotes pro‐inflammatory cytokine activity through nuclear factor kappa‐light‐chain‐enhancer of activated B cells (NF‐κB) signalling.^70^ 5‐Fluorouracil‐based chemotherapy regimens induce skeletal myopathy through p38 and ERK1/2 MAPK activation and impair mitochondrial dynamics.^71^ Thus, it is likely that stress signalling and myopathy would occur with clinically compatible dosing of cytarabine‐based regimens, particularly when multiple induction cycles and consolidation therapy are utilized.

Chemotherapy is also conventionally utilized as a treatment strategy in HCT conditioning programmes, which typically include the non‐specific alkylating agents cyclophosphamide and busulfan. While there are no data regarding the impact of busulfan on skeletal muscle, there are several studies documenting the effect of cyclophosphamide. Cyclophosphamide promotes a negative nitrogen balance, impairs protein metabolism, causes skeletal muscle disorganization, and induces mitochondrial dysfunction: these manifest as exercise intolerance and reduce both MM and BM.^72^, ^73^ Cyclophosphamide also promotes inflammation through increased transcription of NF‐κB and subsequent production of pro‐inflammatory cytokines, which are prominent mediators of skeletal muscle atrophy.^74^ NF‐κB activation is central to the myopathy caused by both chemotherapy and cancer.^5^, ^7^ Identifying the underlying mechanisms of cyclophosphamide‐induced skeletal myopathy requires future exploration as does the impact of emerging CAs for AML treatment, such as multi‐targeted kinase inhibitors, which are known to elicit significant off‐target effects that can induce cardiac and skeletal myopathies.^75^ It is imperative that further research concerning both current and novel drug candidates is conducted to elucidate their relative contribution to cachexia and myopathy induction. Hopefully, this will occur without delay as opposed to the historical paucity of action.

### Myelogenous cytopenia‐related factors

Inextricably, the aim of therapeutic strategies against AML is to nullify non‐functional leukaemic progenitors that have replaced normal myeloid progenitors and inadvertently depleted the blood cells from the myeloid lineage. Paradoxically, chemotherapy induces pan cytopenia leaving the haematological system severely challenged. This is epitomized by the effect of chemotherapy on neutrophils, granulocytes of myeloid lineage. Neutropenia (abnormally low neutrophil count) is a risk factor implicated in the high rates of mortality associated with AML. Neutrophils are master regulators of innate immunity,^76^ and their depletion renders patients immunocompromised and susceptible to opportunistic infections. Febrile neutropenia is a common complication in haematological malignancies, and it accounts for ~11% of patient mortality.^77^ While there are prophylactic drugs to treat arising infections, neutropenia, unlike other forms of cytopenia, cannot be improved through blood transfusions. Moreover, standard neutropenia treatment with granulocyte colony‐stimulating factor has only modest efficacy in AML: while it can reduce neutrophil recovery time, it does not reduce the risk of infections nor improve survivability.^78^ Neutropenic AML patients are confined to wards as a protective environmental prophylactic antibiotic measure.^79^ More severe confinement occurs in patients with vancomycin‐resistant enterococci, an otherwise commonplace colonizer of gut flora, to prevent the spread of infection (and risk of mortality) to other AML patients.^80^ While necessary, these strategies are to the muscular system's detriment: patient inactivity and bed rest promote muscle de‐loading and drive muscle wasting.^81^ This is further complicated by the prevalence of erythropenia, which results in poor tissue oxygenation (i.e. hypoxia), particularly of skeletal muscles. Hypoxia exacerbates inactivity‐related muscle wasting and contributes to muscle fatigue,^82^ which is reported to be the most stressful side effect experienced by cancer (and AML) patients as it incapacitates, reducing QoL.^83^ While further research is required to interrogate the protective role of physical activity against skeletal myopathy and physiological fatigue in AML, exercise has been shown to ameliorate the induction of central fatigue and depression.^84^

Systemic myeloid‐related cytopenia induced by AML and chemotherapy treatment, especially neutropenia and erythropenia, can also directly impact skeletal muscle tissue and drive myopathy. Neutrophils are critical for skeletal muscle remodelling through stimulating monocyte chemotaxis, and subsequently macrophage recruitment, early events in the response to myotrauma.^85^ In the clinical AML setting, myotrauma may result from (i) cellular injury (e.g. oxidative stress and proteolytic system activation) caused by cancer secretions, chemotherapy, and other drugs utilized in standard clinical care and (ii) transient re‐loading after moderate–long periods of de‐loading. Unfortunately, there is limited understanding of the effect of cancer‐related neutropenia on skeletal myopathy, although it is hypothesized that neutropenia blunts the muscle repair response following damage.^85^ Interestingly, monocytes, also derived from the myeloid lineage, may play a larger role in skeletal muscle damage and repair processes compared with neutrophils, because they regulate the recruitment and function of macrophages. Monocytes are involved in the transition of pro‐inflammatory M1‐like macrophages, which remove damaged tissue, to anti‐inflammatory and pro‐fibrotic M2‐like macrophages, which mediate extracellular matrix remodelling and skeletal muscle regeneration.^85^ Recently, it has been demonstrated that chemotherapy reduces the number of skeletal muscle M1‐like macrophages and M1–M2‐like transitional macrophages but has no effect on M2‐like macrophages.^86^ These data highlight that dysregulation of the skeletal muscle immune micro‐environment may contribute to AML and treatment‐related skeletal myopathy and could be a target for prevention/reversal of cachexia.

Erythropenia results in systemic tissue hypoxia, which is clinically managed through blood transfusions until endogenous erythropoiesis recovers. The impact of acute and chronic hypoxia on skeletal muscles in a cachectic environment is relatively uncharacterized. Indeed, erythropenia‐mediated hypoxia causes debilitating physiological fatigue in which a claudication‐like loss of muscle sensation has been reported.^87^ Systemic hypoxia can stimulate molecular adaptations within skeletal muscle through the oxygen‐sensitive target, hypoxia inducible factor‐1α (HIF‐1α).^87^ During acute muscle hypoxia, such as that following intense exercise, HIF‐1α is transiently activated, stimulating an adaptive stress response that clears unhealthy mitochondria and promotes healthy mitochondria biogenesis to mediate recovery.^88^ Conversely, persistent HIF‐1α activation in response to chronic hypoxia impairs bioenergetic efficiency.^88^ Chronic hypoxia is associated with skeletal muscle atrophy and is considered to occur in a fibre type‐dependent manner. Oxidative muscle fibres appear to be more sensitive to hypoxia because their metabolism is dependent on oxygen delivery.^88^ It is an obvious problem for AML patients and is also observed in cancer cachexia, likely as an adaptive stress response to limit oxidative damage or due to tumour‐specific factors.^89^ Chronic hypoxia also supresses mammalian target of rapamycin, mTORC1, a master regulator of protein synthesis and therefore MM, via up‐regulation of *regulated in development and DNA damage 1* (REDD1).^88^ Interestingly, the gene REDD1 is a key transcriptional target of chemotherapy‐induced myopathy.^7^

### Malnutrition and metabolic dysregulation

Historical interpretations of BM wasting in cancer were previously attributed to impaired nutritional status.^90^ Presently, malnutrition remains a key hallmark of cachexia, arising from reduced food intake due to loss of appetite.^6^ During nutrient stress, skeletal muscle undergoes catabolism to unlock stored amino acids for energy production and is magnified during extended periods of hospitalization.^91^ In haematological cancers, ~30% of patients are malnourished.^92^ However, there is dissensus on the incidence of malnutrition in AML. It is estimated that ~15–30% of patients receiving induction chemotherapy, or those in the post‐remission phase, present with malnutrition, which is associated with longer hospital stays and increased level of minimal residual disease, GI toxicity, and risk of mortality.^93^, ^94^ Unsurprisingly, patients who are malnourished prior to HCT are at increased risk of adverse treatment outcomes.^95^ Subsequently, the European Society for Clinical Nutrition and Metabolism established guidelines for malnutrition risk screening and recommendations for intervention, albeit these are poorly adhered to in AML clinical care management.^96^ This may be due to the scarcity of information pertinent to AML. Parenteral feeding has been touted as preferential over enteral feeding due to common oncological challenges (i.e. GI toxicities, tube feeding complications, and absorption difficulties^95^). Conversely, European Society for Clinical Nutrition and Metabolism guidelines recommend enteral feeding due to the lower risk of complications from infections in immunocompromised patients undergoing intense chemotherapy or HCT conditioning programmes.^97^ This highlights a requirement for more extensive clinical investigations to aid clinical care management and decision making through improved risk stratification processes. Delineating AML patients that are wasting due to malnutrition vs. cachexia is imperative because not all malnourished patients are cachectic and thus can respond to nutritional interventions.^98^ In contrast, all cachectic patients are invariably malnourished but typically insensitive to nutrition interventions.^98^

The underlying drivers of malnutrition in AML are poorly understood, but are purported to be driven by chemotherapy‐associated toxicities, particularly GIT‐related side effects.^99^ GIT toxicity is typically attributed to mucositis,^99^ which perturbs food intake behaviours and impairs GIT nutrient absorption. This can exacerbate malnutrition especially in the presence of enteric neuropathy, which is provoked by chemotherapy treatment and results in dysregulated peristalsis, reduced gut motility, and delayed bolus transit time.^100^ Mucositis can also induce complications through neutropenia‐related blood infections arising from intestinal barrier damage. This has been observed following AML chemotherapy induction regimens.^101^ The intestinal barrier also confines the microbiota, a key regulator of GIT homeostasis and integrity: its composition and function is affected by intestinal inflammation, as seen in mucositis, and directly through chemotherapy‐mediated cytotoxicity.^99^ It has recently been shown that reduced microbial diversity, that is, dysbiosis, predicts the risk of infection in AML patients.^102^ Dysbiosis has also emerged as a prognostic factor for mortality in haematological cancer patients undergoing HCT, with increased microbiota diversity associated with higher OS.^103^ Pertinent to skeletal muscle, microbiota diversity has a positive role on physical function and skeletal MM maintenance.^104^ It is also salient in sarcopenia‐related skeletal myopathy.^105^ Thus, in AML where poor prognosis is significantly increased in older adults, it would be interesting to investigate the impact of sarcopenia alongside chemotherapy on microbiota diversity and how this interplay relates to the induction and progression of cachexia.

Metabolic dysregulation is intimately intertwined with malnutrition in cancer cachexia, where a negative energy balance is reflective of disproportionate energy consumption vs. production.^106^ In this context, cancer cell metabolism usurps systemic metabolism, driving the increase in basal metabolic rate.^106^ Cancer cells create metabolic symbiotic relationships with the host whereby systemic glucose metabolism is enhanced to support cell survival and proliferation.^107^ This paradigm has been observed in AML patients, with systemic glucose metabolism progressively enhanced in patients stratified by cytogenetic risk.^108^ Further, AML patients with adverse risk profiles have higher circulating glycolytic metabolites (lactate and pyruvate).^108^ Dysregulation of glucose metabolism negatively affects energy substrate‐rich tissue, such as skeletal muscle, through the proteolytic release of amino acids that are utilized for hepatic gluconeogenesis, and adipose tissue, through the lipolytic release of free fatty acids to be utilized for glycolysis via β‐oxidation.^106^ This is further complicated by insulin resistance, an event that can be potentiated by both AML and chemotherapy.^108^, ^109^ Insulin resistance suppresses the phosphatidylinositol 3‐kinase and protein kinase B signalling pathways and, subsequently, the transcription of downstream effector forkhead box O. These, in turn, enhance the expression of classic proteolytic factors, E3 ubiquitin ligases, muscle RING‐finger protein‐1, and atrogin‐1, a common signalling cascade associated with skeletal muscle atrophy.^110^ In response to reduced insulin‐mediated glucose uptake, skeletal muscle increases the uptake of lipid intermediates, diacylglycerol and ceramide, as a compensatory mechanism to reduce the energy deficit.^111^ However, this mechanism inadvertently promotes intramuscular lipid deposition, that is, myosteatosis, a common feature of primary myopathies that incite progressive muscle dysfunction.^112^ It is important to note that dysregulated metabolism is only one of the contributing factors driving myosteatosis: defective leptin signalling, increased intramuscular pro‐adipogenic progenitor cells, and reduced regenerative potential are also suggested potentiators.^112^ Interestingly, these mechanisms are pervasive in the cachectic phenotype^113^ and are associated with poor survivability in a multitude of cancers.^114^

### Disturbing the inflammatory milieu

Although cachexia induction is a multifactorial phenomenon, it is consistently underpinned by elevated systemic inflammation mediated by an increased pro‐inflammatory to anti‐inflammatory cytokine ratio.^6^ The central nervous system is a crucial regulatory target of cachexia induction and progression through its role in the recognition of inflammatory cytokines as signallers of the acute illness response.^115^ Subsequently, the central nervous system can evoke systemic catabolism through hypothalamic–pituitary–adrenal axis activation, which contributes to fatigue, malnutrition, and BM and MM loss.^6^ The utility of pro‐inflammatory biochemical markers has emerged through specific diagnostic tools, including the (i) Glasgow prognostic score (GPS), which utilizes C‐reactive protein and albumin to diagnose cachexia^116^ and, and (ii) CAchexia SCOre (CASCO), which incorporates the pro‐inflammatory cytokine interleukin‐6 in its criteria panel for quantitative staging of cancer patients across the cachexia continuum.^117^ While these strategies are a positive step towards improved cachexia identification, especially because it is typically under‐diagnosed, one disadvantage of these approaches is that the inflammatory milieu is heterogeneously modulated by different types of cancer and typically involves complex and specific interactions between the cancer and host.^118^ Solid tumour cancers release mediators (i.e. a secretome), including pro‐inflammatory cytokines, which promote systemic inflammation and induce cachexia.^118^ In haematological malignancies like AML, the role of the secretome on cachexia induction has been largely ignored despite evidence suggesting that inflammatory mediators promote AML disease progression.^119^ Freire *et al*. were the first to demonstrate the AML secretome releases cachexia‐inducible factors (CIFs) that contribute to cachexia induction and are associated with an increased risk of mortality.^118^ Because AML is a heterogeneous disease with multiple subtypes, it would be imperative to stratify the datasets surrounding CIFs by cytogenetic mutations to identify translational pipelines specific to AML. Future investigations in this area will enrich the academic utility of the Freire *et al*., paper, which exhaustively predicted ~613 dysregulated genes from the AML secretome.^118^ There is limitless potential for future studies stemming from this data given that the AML‐specific cachexia field is largely untouched.

Future studies investigating AML‐derived CIFs will also have the potential to aid development of screening strategies for AML‐specific cachexia risk stratification using biomarkers. Current biomarkers are overtly non‐specific to cancer type and do not delineate the general ratio between pro‐inflammatory and anti‐inflammatory cytokines.^64^ However, biomarker strategies for cachexia screening may still be useful in haematological malignancies such as AML, given their current lack of utility. Clinical trials are now required to evaluate their efficacy, alongside investigating other emerging biomarkers that can identify both MM loss and changes to the inflammatory cytokine ratio.^120^

## Perspectives and conclusions

Historically, cachexia, and its clinical repercussions, has been overlooked in haematological malignancies, like AML, focussing instead on solid tumour cancers that present with more profound body and muscle wasting.^6^ Nevertheless, AML research is typically partisan towards risk stratification associated with patient cytogenetics: this has been relatively successful, particularly in the AML subtype, acute promyelocytic leukaemia, through the identification of a novel gene therapy, which proffers improved survivability.^121^ Despite the clinical utility of risk stratification based on cytogenetics, patients in each cytogenetic risk group remain prognostically heterogeneous.^18^ Thus, further stratification strategies are required. These could include (i) scoring criterion related to skeletal muscle health such as CT‐derived SMI or (ii) cachexia diagnostic tools, GPS, and CASCO, to better inform clinical decision making and improve patient survivability. While serum biomarker scoring systems like GPS and CASCO are relatively easy to implement and interpret within the clinical AML setting during routine pretreatment blood work‐up, CT‐derived SMI quantitation is labour intensive, requires interpretive expertise, and is not routinely performed for AML work‐up. Emerging automated technologies, such as deep learning artificial intelligence, will facilitate implementation and integration of CT‐derived SMI into clinical AML care.^122^ The use of novel AML‐derived CIFs^118^ as personalized biomarkers for cachexia risk stratification is also not outside the realm of possibility given that AML is already a frontier for personalized medicine.

While the identification of cachexia in AML is an exciting opportunity for risk stratification, it also represents a detrimental co‐morbidity that requires therapeutic intervention. Thus, strategies that protect skeletal muscle health could play a key role in mitigating AML‐related and treatment‐related toxicities, with the central goal of improving patient eligibility for HCT. Given the heterogeneity of potential contributing factors to skeletal myopathy induction in the AML setting, multifactorial strategies will likely be required for efficacy. The Multimodal‐Exercise, Nutrition and Anti‐inflammatory medication for Cachexia (MENAC) Phase III trial has shown promise in treating cachexia arising from other cancer types.^123^ Indeed, more research is required surrounding novel strategies to promote ambulation and load bearing during extended periods of hospitalization, particularly those that acknowledge the environmental challenges of ward and/or room confinement experienced by AML patients. However, before multifaceted interventions like the MENAC trial can be translated into the AML setting, it is imperative to consider the T.A.R.G.E.T. approach, which highlights the need to initially pursue strategies that promote teaching, awareness, and recognition of the effect of AML and its treatments on skeletal muscle to further establish this emerging field.^124^

## Conflict of interest

None declared.
